# Improving the primary care physicians’ decision making for fibromyalgia in clinical practice: development and validation of the Fibromyalgia Detection (FibroDetect®) screening tool

**DOI:** 10.1186/s12955-014-0128-x

**Published:** 2014-10-24

**Authors:** Ralf Baron, Serge Perrot, Isabelle Guillemin, Cayetano Alegre, Carla Dias-Barbosa, Ernest Choy, Hélène Gilet, Giorgio Cruccu, Jules Desmeules, Joëlle Margaux, Selwyn Richards, Eric Serra, Michael Spaeth, Benoit Arnould

**Affiliations:** Division of Neurological Pain Research and Therapy, Christian-Albrechts University, Kiel, Germany; Internal Medicine Department and Pain Center, Paris Descartes University, INSERM U987, Paris, France; Mapi HEOR & Strategic Market Access, 27, Rue de la Villette, 69003 Lyon, France; Rheumatology, Hospital Universitari Valle d’Hebron, Barcelona, Spain; Department of Rheumatology, Cardiff University, Cardiff, UK; Department of Neurology and Psychiatry, La Sapienza University, Rome, Italy; Clinical Pharmacology and Toxicology & Multidisciplinary Pain Center, Geneva University Hospital, Geneva, Switzerland; Rheumatology and Physical Medicine Department, Erasme Hospital, Brussels, Belgium; Poole Hospital NHS Trust, Rheumatology, Poole, UK; Pain Unit, Amiens University Hospital, Amiens, France; Center for Clinical Rheumatology, Gräfelfing, Germany

**Keywords:** Fibromyalgia, Primary health care, Screening tool, Early diagnosis, Decision making, FibroDetect

## Abstract

**Background:**

Fibromyalgia diagnosis is a challenging and long process, especially among primary care physicians (PCPs), because of symptom heterogeneity, co-morbidities and clinical overlap with other disorders. The purpose was to develop and validate a screening tool in French (FR), German (DE) and English (UK) to help PCPs identify patients with fibromyalgia.

**Methods:**

The FibroDetect questionnaire was simultaneously developed in FR, DE and UK based on information obtained from a literature review, focus groups conducted with clinicians, and face-to-face interviews with fibromyalgia patients (FR, DE and UK, n = 23). The resulting tool was comprehension-tested in patients with diagnosed or suspected fibromyalgia (n = 3 and n = 2 in each country, respectively). Acceptability and applicability were assessed and the tool modified accordingly, then assessed in clinical practice. A scoring method was created using an iterative process based on statistical and clinical considerations with American College of Rheumatology + (ACR+) patients and ACR– patients (n = 276), and validated with fibromyalgia and non-fibromyalgia patients (n = 312).

**Results:**

The FibroDetect included 14 questions assessing patients’ pain and fatigue, personal history and attitudes, symptoms and impact on lives. Six questions were retained in the final scoring, demonstrating satisfactory discriminative power between ACR + and ACR- patients with area under the Receiver Operating Characteristic curve of 0.74. The predictive accuracy of the tool increased to 0.86 for fibromyalgia and non-fibromyalgia patient detection, with a sensitivity of 90% and a specificity of 67% for a cut-off of 6 on the score.

**Conclusions:**

The FibroDetect is a self-administered tool that can be used as a screening classification surrogate to the ACR criteria in primary care settings to help PCPs detect potential fibromyalgia patients among a population complaining of chronic widespread pain.

## Introduction

Fibromyalgia is a chronic condition associated with widespread pain, fatigue and tender points [1,2]. A recent European survey conducted in France, Germany, Italy, Portugal and Spain estimated a prevalence ranging from 1.4% (France) to 3.7% (Italy) [3]. The condition affects women more commonly than men and the incidence increases with age [4,5].

The American College of Rheumatology (ACR) criteria provided guidelines for researchers to define fibromyalgia [2] that are used extensively by doctors in everyday practice to diagnose fibromyalgia. To be diagnosed with fibromyalgia, one must have experienced widespread pain for at least 3 months and have a minimum of 11 out of the 18 specified points on the body that are painful under relatively mild, firm pressure (<11 points classified ACR-; > 11 points classified ACR+); the other diseases that could mimic fibromyalgia symptoms should be excluded [2]. More recently, in an attempt to address the subjectivity in performing these tender point exams and thus simplify diagnosis in primary and specialty care, preliminary diagnostic criteria that eliminates the requirement for a physical or tender point exam have been proposed by the ACR [6]. These updated criteria consist of both the widespread pain index (WPI) and the symptom severity (SS) scale that are to be completed by the clinicians.

Despite these attempts, fibromyalgia diagnosis is challenging for several reasons, including the high heterogeneity of symptoms; the many co-morbidities (i.e., digestive, psychological and cognitive dysfunction) in these patients; and the overlap of symptoms associated with other musculoskeletal, rheumatologic and psychiatric disorders, or pain-causing conditions like neuropathic pain [1,2,7–10]. Even though clinicians’ awareness and scepticism about fibromyalgia have favourably changed in the last years, fibromyalgia condition is still under diagnosed.

Although the ACR criteria are widely used and sensitive, they remain problematic for health professionals, especially primary care physicians (PCPs) who rarely or improperly use them [11,12] to establish a fibromyalgia diagnosis [11,12]. In practice, PCPs are often the first health professionals fibromyalgia patients consult because of their poor quality of life. They are recommended to use ACR criteria in conjunction with the patients’ medical history, symptoms and co-morbidities, and the results of biological and laboratory exclusion tests [13]. Consequently, patients are likely to undergo multiple consultations before receiving a positive diagnosis and adequate care management [14]. Incorrect diagnosis is also very frequent. Delayed or misdiagnosis, along with the condition itself have major impact on patients’ emotional state and quality of life, as well as on societal and health care costs [15,16]. Therefore, a simple questionnaire for PCPs that is capable of translating patients’ complaints and experience into clues to potential fibromyalgia would be highly valuable. A review of the existing questionnaires showed that these either focus on a specific aspect, domain or symptom of fibromyalgia [7,17–22], or have not been adapted for use in clinical practice [23]. The content of the Multidimensional Health Assessment Questionnaire (MDHAQ) makes it an interesting candidate; however, its complex scoring restrains its use in clinical practice [24]. More recently, the Fibromyalgia Rapid Screening Tool (FiRST) and the Fibromyalgia Diagnostic Screen have been designed to identify fibromyalgia in daily practice and clinical research [25,26]. Both require further work to determine their performances in real-life conditions by PCPs. The promising discriminative value of the FiRST was determined based on well diagnosed patients (fibromyalgia versus rheumatoid arthritis, osteoarthritis and ankylosing spondylitis), and thus may not reflect real-life, with undiagnosed patients consulting with pain and/or fatigue complaint [26]. As for the Fibromyalgia Diagnostic Screen, the tool lies on clinician testing, in particular the tender points [25], which could make the tool not practical and not reliable for use by PCPs. Finally, the completion by clinicians of the updated ACR criteria may introduce a bias in patients’ responses, and may therefore be not as accurate as when patients directly report and describe their experience and feeling.

To answer the need for a reliable and specific tool that assists PCPs in screening for fibromyalgia in their day-to-day practice, we developed the FibroDetect tool. Its development followed a standardized methodology, which should ensure its acceptability by the scientific community and suitability for use in clinical practice [27]. The validation study resulted in the definition of a scoring method and a threshold allowing for identification of potential fibromyalgia patients through the discrimination between ACR– and ACR + patients. This paper reports the process for simultaneous development and validation of FibroDetect.

## Methods

### Ethics

The project was performed in accordance with Good Clinical Practices and in compliance with local regulatory requirements. The appropriate national authorities and institutional review boards approved the project before project commencement. Each patient gave informed consent.

The study has been approved by:

- UK (pilot study): NHS National Research Ethics Services Bournemouth and Poole; Dorset Primary care Trust; Hampshire Community Health Care; Stockport Research Ethics Committee.
- DE: Ethics from the study national coordinator (Ralf Baron), i.e. ethics from Kiel Medizinische Fakultät der Christian-Albrechts Universität zu Kiel. This approval has been notified to the following landers: Landesärztekammer Baden-Württemberg; Bayerischen Landesärztekammer; Ärztekammer Hamburg; Uni Greifswald; Ärztekammer Niedersachsen; Ärztekammer Nordrhein; Ärztekammer Saarlandes; Sächsische Landesärztekammer; Ärztekammer Schleswig-Holstein), who approved.
- FR: National Commission for Data Protection and Liberties (CNIL) and Committee for personal protection (CPP) from Ile de France, Hôtel Dieu; 75181 Paris Cédex 04.

### International Working Group

A European multidisciplinary group, representative of the several clinical fields involved in the management of fibromyalgia, was convened. It was composed of pain specialists (n = 3), rheumatologists (n = 4), a psychiatrist, a neurologist and a pharmaco-toxicologist working in a pain centre. It also included experts in patient questionnaire development methodology (n = 3). The experts were involved throughout the development and validation process of the FibroDetect tool. They provided clinical expertise and identified key issues in fibromyalgia detection that are seen in practice.

### Development phase

#### Development of the conceptual model and test version of the FibroDetect

A literature review was performed to collect information on the symptoms, screening and diagnosis, influencing and triggering factors that characterize fibromyalgia. In parallel, focus groups with PCPs were conducted in France (n = 7) and in the UK (n = 8) to explore their experience of fibromyalgia and to learn about their expectations and unmet needs in their practices. Focus groups previously conducted with German PCPs, pain specialists and rheumatologists completed the data.

Following the literature review and PCP interviews, face-to-face interviews with 23 patients (n = 10 in France and in Germany, n = 3 in the UK) were performed by psychologists to explore their experiences and perceptions of fibromyalgia, their medical history relating to fibromyalgia, and their description of pain. Eligible patients had a recent diagnosis of fibromyalgia (≤3 months) with widespread pain as the main complaint.

Focus groups and interviews were performed using a semi-structured interview guide and a non-directive technique so that participants answered questions spontaneously, without the bias of the interviewer intervention [28]. Transcripts from the focus groups were analysed using a thematic analysis approach, extracting information related to the research question (i.e., to help PCPs identify fibromyalgia in their practice) and organising it into concepts and sub-concepts.

**Figure 1:**
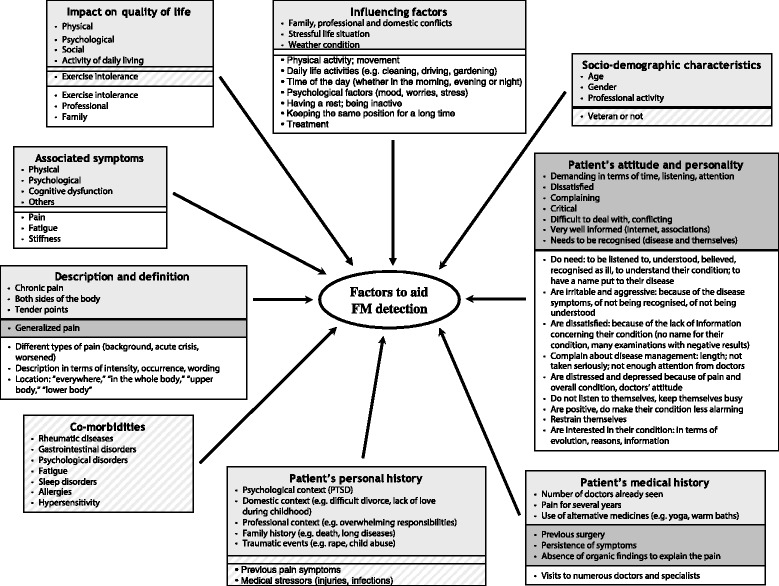
**Conceptual model of factors that could help primary care physicians in the detection of fibromyalgia in practice.** Model developed based on literature review, clinicians’ focus groups and patients’ interviews. Boxes with no colour, information retrieved from patients interviews only; boxes in light grey, information retrieved from the literature review, focus groups and patient interviews; hatched boxes, information retrieved from the literature review only; boxes in dark grey, information retrieved from PCPs focus groups only.

Factors identified from the literature review, clinicians’ focus groups and patient interviews that may help in the detection of fibromyalgia were organised into a conceptual model of the concepts and sub-concepts relative to the research question. No new concepts and information were identified from further Belgian and Swiss interviews (n = 6; interviews conducted in French), confirming saturation of our data. The resulting conceptual model was discussed, amended and agreed upon by the experts of the working group (see Figure 1).

Using patients’ own words, items covering each of the concepts and sub-concepts of the final conceptual model were generated simultaneously in French, German and UK English, along with response choices, instructions and layout of the screening tool. The resulting test version of the tool was reviewed by the international working group to ensure its clinical relevance.

#### Assessing the test version of the FibroDetect

The test version was tested with another 15 patients in France, Germany and the UK (5 patients per country). Psychologists conducted face-to-face comprehensive testing interviews. The purpose was to ensure comprehensiveness, clarity, cultural relevance and appropriateness of the questionnaire for the patients. Eleven patients were diagnosed with fibromyalgia and 4 patients were experiencing unexplained chronic pain but not yet diagnosed with fibromyalgia. Cognitive interviewing methodology was used, allowing researchers to understand the cognitive processes involved when respondents answered questions [28]. Based on patients’ comments, the test version was revised. Upon approval from the experts, the revised test version was harmonized among the three languages and checked for linguistic equivalence by a linguistic expert. A pilot version was obtained in the three languages.

#### Testing the pilot version of the FibroDetect in real-life conditions

The acceptability of the tool, in terms of relevance, ease of use and applicability by PCPs in their clinical practice, was assessed using the PRAgmatic Content and face validity-Test^©^ (PRAC-Test^©^) [29] during a cross-sectional, observational, multicentre study conducted in the UK, Germany, and France with PCPs. Fourteen PCPs participated in the study, recruiting a total of 34 patients (16 in France, 15 in Germany and 3 in the UK). Eligible PCPs had to be familiar with fibromyalgia. Patient selection criteria were the same as for the comprehension testing.

Based on comments and suggestions from PCPs, the tool was further revised. The final revision, approved by the experts, resulted in the version of the FibroDetect ready for psychometric validation.

### Validation phase

#### Study design and population

**Figure 2:**
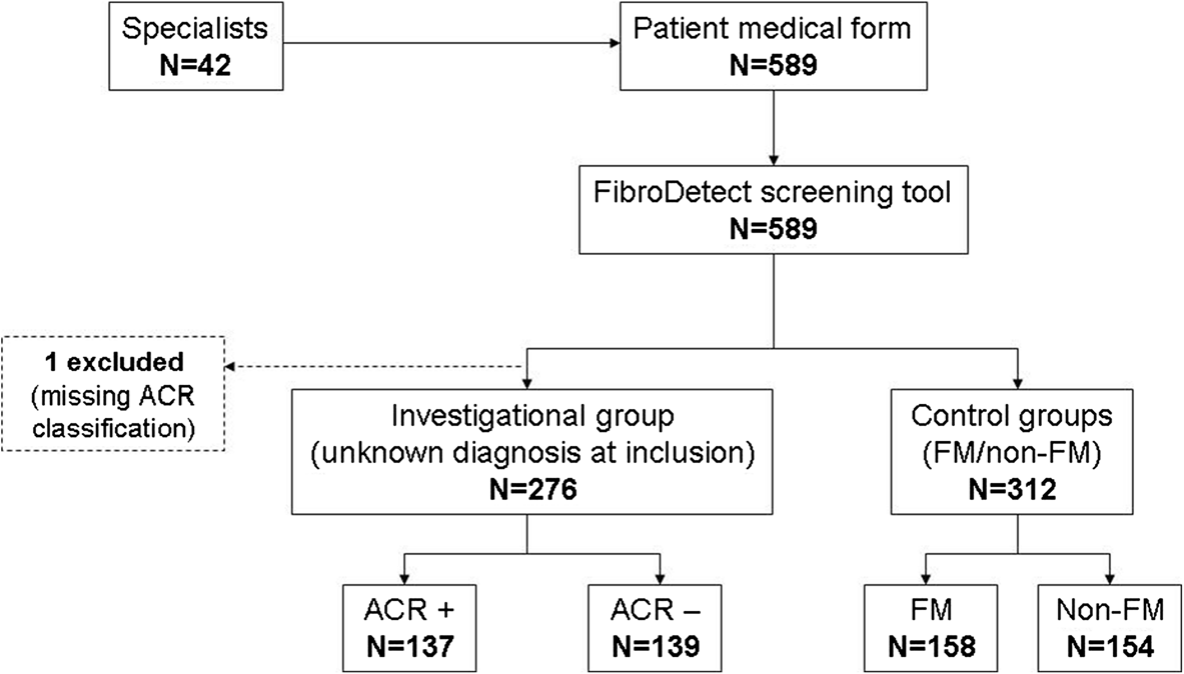
**Flow chart of the populations for the validation study.** ACR, American College of Rheumatology; FM, fibromyalgia.

A cross-sectional, observational and multicentre study was conducted in France and Germany. ACR 1990 criteria were used as the gold standard for detection of fibromyalgia [2]. Because ACR criteria are seldom used and are sometimes misused by PCPs, the validation study took place in secondary care settings. Patients were recruited by rheumatologists, pain specialists and neurologists experienced in the diagnosis and management of patients with fibromyalgia; these were classified into one investigational group and two control groups (see Figure 2). Patients whose ACR were not determined (ACR-not determined) constituted the investigational group and corresponded to the target population of the tool; this group included patients with chronic widespread pain for which no diagnosis was established at the time of their visit to the specialist. Fibromyalgia and non-fibromyalgia groups constituted the control groups. The fibromyalgia group corresponded to patients diagnosed with fibromyalgia at least 6 months prior to inclusion; the non-fibromyalgia group included patients diagnosed with a confirmed disease for chronic widespread pain other than fibromyalgia (localized musculoskeletal pain, common localized low back pain, osteoarthritis, inflammatory arthritis, lupus, multiple sclerosis, peripheral neuropathy, post-herpetic neuralgia, cancer pain, migraine/chronic tension headache, complex regional pain syndrome). Recruiting physicians were asked to classify their ACR-not determined patients as ACR + or ACR- after clinical examination and FibroDetect completion, based on the ACR criteria and their own opinion and knowledge of the patient.

#### Item reduction and creation of the discriminant model for the classification of ACR patients

The discriminant model (i.e., the combination of FibroDetect items allowing separation of patients), was created with the investigational group (ACR + and ACR– patients). Partial least squares discriminant analysis (PLS-DA) was performed successively on different combinations of items of the screening tool to determine the items that best separated ACR + patients from ACR– patients and to create the FibroDetect scoring method. PLS-DA is a multivariate classification method that aims to associate a dependent variable block Y (here, the ACR classification) to a covariate block X (here, the FibroDetect items). PLS regression combines features from principal component analysis (PCA) and multiple regression, and is able to account for multicollinear variables, incomplete data, and a larger number of variables in comparison to the number of observations [30,31]. Items to be kept in the successive models were selected from an iterative process based on 1) the percentage of missing responses to each item; 2) the odds ratio with 95% confidence interval for each individual item, evaluating its ability to discriminate between ACR + and ACR– classifications; 3) the variable of importance (VIP) of individual items resulting from PLS-DA, with a VIP criterion higher than 1 generally considered of major importance in the discriminant model [32]; 4) relevance from clinicians’ and patients’ perspectives. In addition, the ability of each model (combination of items) to separate ACR + from ACR– patients was evaluated using the Area Under (AUC) the Receiver Operating Characteristic (ROC) Curve. The higher the AUC, the better the prediction; an area value of 0.5 means predictions were not better than random guessing [33].

#### Definition of a threshold for classification of ACR patients

Once the scoring method was created, a score threshold was defined to optimize the classification of patients at the individual level and ensure that a maximum number of ACR + patients were properly classified. This should maximize the probability for ACR + patients to be correctly classified as ACR + patients (i.e., the sensitivity) while limiting the probability for ACR– patients to be incorrectly classified. The score threshold was determined for the best discriminant model obtained and was selected to correspond to a sensitivity of 95% with a specificity (i.e., the probability for ACR– patients to be correctly classified as ACR– patients) of at least 50%.

#### Testing the ability of the FibroDetect to differentiate patients with fibromyalgia from those without fibromyalgia (non-fibromyalgia)

As part of the validation process of the FibroDetect, the discriminant model and threshold defined with ACR + and ACR– patients were applied to the control group population to evaluate their ability to separate fibromyalgia patients from non-fibromyalgia patients.

#### Statistical software and statistical threshold

The threshold for statistical significance was p < 0.05 for each test. An odds ratio with a 95% confidence interval excluding the value 1 indicated that the item was significantly able to discriminate between groups of patients.

Statistical analyses were performed with SAS software for Windows (Version 9.2, SAS Institute, Inc., Cary, NC, USA). PLS-DA was performed using SIMCA Software (SIMCA-P Version 10.0.4.0, Copyright© 1993–2002 Umetrics AB).

## Results

### Development of the FibroDetect

#### Conceptual model of the factors that may aid PCPs in the detection of potential fibromyalgia and test version of the FibroDetect

The conceptual model, developed from the literature, PCP and patient findings, comprised nine concepts that were considered by the clinical experts to be factors that could help PCPs in the detection of fibromyalgia. These factors were: “description and definition of fibromyalgia,” “associated symptoms,” “co-morbidities,” “impact on patients’ lives,” “influencing factors,” “patients’ socio-demographic characteristics,” “patients’ attitudes and behaviours towards fibromyalgia,” “patients’ medical history” and “patients’ personal history” (Figure 1). After item generation, the test version of the screening tool contained 14 questions, including a silhouette for patients to indicate location of pain.

### Validity

#### Development of the pilot version of the FibroDetect: content validity

The test version of the FibroDetect was very well accepted by patients. The majority of the patients found the questions and item content to be relevant and understandable and the format of the tool appropriate. Items and response choices that were found too confusing, overlapping, not capturing the targeted concept appropriately or not understood correctly were reformulated, reworded or reorganized. One question was added that allows patients to describe their current health status; two other questions were combined. Upon experts’ approval, the revised version of the screening tool was harmonized among languages and checked for linguistic equivalence. The pilot version of the tool was named Fibromyalgia Detection screening tool (FibroDetect), and was finalised in UK English, French and German.

#### Pilot testing of the FibroDetect in real-life conditions: face validity

Analysis of the PRAC-Test completed by the PCPs (n = 14) supported the overall good acceptability of the screening tool by practicing PCPs. There was a strong concordance between the objectives of the tool and PCPs’ feedback. They found it useful for making their decision and for improving communication with their patients; they found the information collected via the tool relevant. Six PCPs (n = 6) suggested shortening and simplifying questions as much as possible.

The pilot version of the tool was further revised according to PCPs’ comments and suggestions. After a final check for linguistic equivalence and harmonisation across the three language versions, the FibroDetect tool was ready for the validation study. This version was a 4-page leaflet (11.7 inches high × 3.94 inches wide), containing 14 questions, including one silhouette. Its structure and content are described in Table 1.

**Table 1:** **Structure and content of the FibroDetect screening tool (version for validation study)**

**COVER PAGE:** to invite the appropriate patients to complete the screening tool	
• Attention-capturing sentences	
**INSIDE PAGE:** to allow the patients to describe their pain, fatigue and other symptoms	
Question 1^*^	• Location of the pain (body diagram to complete)
Question 2	• Frequency of the pain
Question 3	• Description of the pain (list of sensations/perceptions)
Question 4	• Frequency of tiredness
Question 5	• Impact of physical effort on tiredness
Question 6	• Symptoms patients experienced that are associated with their condition (list of symptoms)
**INSIDE PAGE:** to capture the link between patients’ condition and life, and the impact of fibromyalgia on their everyday life, personality and attitude	
Question 7	• Impact of situations on patients’ condition (list of physical, psychological and external situations)
Question 8	• Impact of patients’ condition on their everyday life areas (list of life areas)
Questions 9 and 10	• Assessment of traumatic or stressful events in patients’ life
Question 11	• Attitude and behaviour of patients faced with their condition (list)
Question 12	• Statements about the disease
Question 13	• Extent to which patients recognise themselves in the questions being asked
Question 14	• Content of the tool allowing report of current health status

*To facilitate the use of and optimise the information resulting from answers collected for the silhouette, the front and back silhouettes were divided into 16 body areas, and one item for the number of areas indicated by the patient.

#### Item reduction and creation of the discriminant model for the classification of ACR patients

A total of 589 patients were recruited into the validation study (Table 2). The classification of the study populations is summarised in Figure 2. All 589 patients recruited by specialists (42 specialists recruited at least 1 patient each) completed the FibroDetect questionnaire. Among these patients, one subject had a missing final ACR classification and was thus not included in the investigational group.

**Table 2:** **Socio-demographic and clinical characteristics of patients who participated in the validation study (N = 588)**

**Variable**		**Investigational group**		**Two control groups**	
		**ACR + (N = 137)**	**ACR– (N = 139)**	**FM (N = 158)**	**Non-FM (N = 154)**
**Country - N (%)**	**Germany**	45 (32.8)	62 (44.6)	41 (25.9)	36 (23.4)
	**France**	92 (67.2)	77 (55.4)	117 (74.1)	118 (76.6)
**Age (years)**	**N**	137	139	158	154
	**Mean (SD)**	51.5 (11.0)	52.3 (14.0)	52.0 (11.6)	55.3 (14.6)
	**Median (Q1 - Q3)**	52.0 (46.0 - 58.0)	52.0 (43.0 - 61.0)	53.0 (44.0 - 59.0)	55.0 (46.0 - 65.0)
	**Min – Max**	19.0 - 87.0	19.0 - 89.0	25.0 - 87.0)	18.0 - 83.0)
**Gender - N (%)**	**Female**	118 (86.1)	114 (82.0)	145 (91.8)	109 (70.8)
**Time since first chronic widespread pain (years)**	**N**	129	137	152	-
	**Mean (SD)**	6.1 (6.8)	5.5 (7.7)	9.9 (9.6)	
	**Median (Q1 - Q3)**	4.0 (1.0 - 9.0)	3.0 (1.0 - 5.0)	7.0 (4.0 - 13.0)	
	**Min – Max**	0.0 - 36.0	0.0 - 42.0	0.0 - 62.0	
**Number of doctors already visited for pain and/or fatigue - N (%)**	**First doctor**	7 (5.1)	6 (4.3)	4 (2.5)	-
	**2-5**	106 (77.4)	113 (81.3)	95 (60.1)	
	**> 5**	21 (15.3)	20 (14.4)	52 (32.9)	
**Number of tender points - N (%)**	**< 11**	18 (13.1)	124 (89.2)	-	-
	**≥ 11**	119 (86.9)	15 (10.8)		

The percentage of missing responses to the FibroDetect items ranged from 0.0% to 14.4%. The highest percentages of missing responses were found for items of question 11 (coping attitudes), and for items of question 7 (influencing factors), regardless of the investigational or control group. On average, patients had 1 missing item for each group. More than two-thirds of patients had no missing item (67% for ACR + patients, 66% for ACR– patients, 73% for fibromyalgia patients and 68% for non-fibromyalgia patients).

The discriminant model was created from an iterative process, starting with the 14-question long original FibroDetect. Discriminant models were successively tested. For each model, the ROC curve was drawn and evaluated in terms of ability to separate ACR + patients from ACR– patients using the AUC.

**Figure 3:**
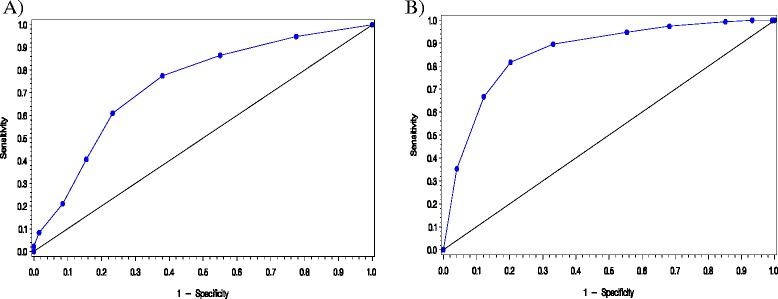
**ROC curve of the final FibroDetect discriminant model; A: Investigational group (ACR + and ACR-); B: Control groups (FM + and FM-).** Sensitivity: probability for ACR+ or FM+ patients to be correctly classified as ACR+ / FM +; 1 – Specificity: probability for ACR– or non-fibromyalgia patients to be incorrectly classified; Diagonal: AUC = 0.5, i.e., predictions are not better than random guessing AUC under the ROC curve: Area Under the Receiver Operating Characteristic curve; ACR, American College of Rheumatology; FM, fibromyalgia.

Item coding was simplified based on item distribution, content and clinical considerations. The best performing and simplest model found to separate ACR + from ACR– patients included questions 1 to 6 (about physical evaluation) and question 13 (about patient recognizing self in the questions asked in the questionnaire) (Table 3). Further PLS-DA were performed on models combining clinical variables collected at the doctor visit with FibroDetect questions or using weighted scores; this did not substantially increase the ability of the model to discriminate between ACR + and ACR − patients. The FibroDetect score corresponding to the discriminant model described above was calculated as the sum of the 9 item scores, thus ranging from 0 to 9. The FibroDetect score was calculated only if all 9 items were completed; otherwise the score was set to missing. The AUC corresponding to the model was of 0.74, with a sensitivity of 77% and a specificity of 61% for a score threshold of 6 (Figure 3).

**Table 3:** **Final discriminant model based on modified FibroDetect items**

**Question**		**Response choices**	**Coding**
**1**	**At least one body part of upper body ticked (head, neck, shoulders)**	Yes	1
		No	0
	**At least one body part of upper limb ticked (right and left arms)**	Yes	1
		No	0
	**At least one body part of lower limb ticked (right and left legs)**	Yes	1
		No	0
**2**	**Frequency of the pain**	Every day or Almost every day	1
		Some days	0
**3**	**At least 3 kinds of pain ticked** ^*^	Yes	1
		No	0
**4**	**Frequency of tiredness**	Every day	1
		Some days or Never	0
**5**	**Impact of physical effort on tiredness**	Much more tired	1
		Slightly more tired or No difference	0
**6**	**At least 7 symptoms ticked** ^**^	Yes	1
		No	0
**13**	**Extent to which patients recognize themselves in the questions being asked**	Absolutely	1
		A little or Not at all	0

*Among 8 kinds of pain: muscle aches, cramps, pins and needles, stabbing, shooting pain, burning, pulling and pain that moves from one place to another.

**Among 17 symptoms: stiffness, headaches, tiredness, tiredness on waking up, exhaustion, insomnia, disrupted or disturbed sleep, sensitive to cold or heat, sensitive to smells, sensitive to noise, sensitive to touch, problems remembering things, problems concentrating, nausea, diarrhoea, constipation and problems with urination.

Questions 7 to 12 and question 14 of the original FibroDetect, even though not included in the calculation of the scores, were kept within the questionnaire.

#### Definition of the threshold

Based on the sensitivity and specificity of the FibroDetect score (Figure 3), three thresholds were defined: ≤ 3, 4–5, and ≥ 6 (Table 4). According to these thresholds, less than 9% of ACR + patients and about a quarter of ACR– patients had a score ≤ 3, 15% of ACR + patients and about 40% of ACR– patients had a score of 4 or 5, and more than three-quarters of ACR + patients and about 40% of ACR– patients had a score ≥ 6.

**Table 4:** **Description of FibroDetect score and thresholds**

**FibroDetect score**		**Investigational group**				**Control group**			
		**ACR + (N = 137)**		**ACR– (N = 139)**		**FM (N = 158)**		**Non-FM (N = 154)**	
**FibroDetect score**	**N**	129		133		153		148	
	**Mean (SD)**	6.8 (1.9)		5.1 (1.9)		7.6 (1.5)		4.7 (2.1)	
	**Median (Q1 - Q3)**	7.0 (6.0 - 8.0)		5.0 (4.0 - 6.0)		8.0 (7.0 - 9.0)		5.0 (3.0 - 6.0)	
	**Min – Max**	2.0 - 9.0		1.0 - 9.0		2.0 - 9.0		0.0 - 9.0	
**Thresholds** ^**1**^	**≤ 3 - N (%)**	11	(8.5)	28	(21.1)	4	(2.6)	47	(31.8)
	**4-5 - N (%)**	19	(14.7)	53	(39.8)	12	(7.8)	52	(35.1)
	**≥ 6 - N (%)**	99	(76.7)	52	(39.1)	137	(89.5)	49	(33.1)

^1^Missing data were not included in the calculation of percentages.

#### Testing the ability of the FibroDetect to differentiate patients with fibromyalgia from those without fibromyalgia (non-fibromyalgia)

When applied to the fibromyalgia and non-fibromyalgia control group, the FibroDetect discriminant model (composed of 6 questions) resulted in an AUC of 0.86, indicating a good ability of the model to separate fibromyalgia from non-fibromyalgia patients (Figure 3). According to the thresholds defined on the FibroDetect score, 90% of fibromyalgia and 33% of non-fibromyalgia patients had a score ≥ 6, while 3% of fibromyalgia and 32% of non-fibromyalgia patients had a score ≤ 3; 8% of fibromyalgia and 35% of non-fibromyalgia patients had a score of 4 or 5 (Table 4).

## Discussion

The FibroDetect tool was developed to meet the need of PCPs for a specific tool to help them screen for potential fibromyalgia patients in their routine practice. It is available in French, German and UK English and is validated in French and German. The development phase of the tool followed a standardised methodology [27] as well as regulatory authorities’ recommendations, with involvement of all parties who are ultimately targeted by the tool, i.e., patients and PCPs [34,35]. Altogether, these ensure the credibility, robustness and acceptability of the FibroDetect as a scientifically sound screening tool; the involvement of a group of multidisciplinary fibromyalgia experts throughout the work reinforces the clinical relevance and appropriateness of the tool.

The validation study then allowed the finalization of FibroDetect, and consisted in item selection and calculation of the score of the discriminant model and in the assessment of the ability of this discriminant model to differentiate patients with ACR + from those with ACR–. The optimal discriminant model of FibroDetect, in terms of simplicity of the score calculation and performances included the questions related to the physical burden of fibromyalgia and the question about the patient’s self-recognition based on the information in the questionnaire. A score ranging from 0 to 9 could be easily and quickly computed from responses given by patients to these questions. Patients with a FibroDetect score ≤ 3 are unlikely to be ACR + patients, and should thus not be referred to a fibromyalgia specialist; patients with a FibroDetect score of 4 or 5 may require further evaluation to decide whether they should be referred to a fibromyalgia specialist; patients with a FibroDetect score ≥ 6 are likely to be ACR + patients, and should thus be referred to a fibromyalgia specialist. The AUC of the model was 0.74, with a sensitivity of 77% and a specificity of 61% for a score threshold of 6. At first, these values may appear limited when compared to other tools recently developed, e.g., FiRST [26] or Fibromyalgia Diagnostic screen [25]. However, the FibroDetect model was created and validated with patients for whom there was a diagnosis challenge (they were classified as ACR + or ACR– after inclusion and clinical examination), unlike the FiRST study which used patients who already passed the difficult process of diagnosis [12]. In this particular context, one could assume that an AUC of 0.74 reflected very good ability to discriminate patients with no diagnosis established at inclusion but with chronic pain and fatigue as complaint. In real life contexts, these symptoms characterise the patients who consult PCPs. When applied to diagnosed fibromyalgia and non-fibromyalgia patients, the AUC of the FibroDetect tool reached 0.86, approaching the value of the FiRST [26], with a sensitivity of 90% and a specificity of 67% for a threshold of 6, thus confirming the good predictive property of the FibroDetect in cases where diagnosis is pre-established. The design of the FibroDetect project, unlike the FiRST, is likely to allow predictive values to be generalised to the targeted patient population.

Findings from both the qualitative phase of this project and from literature [18,36–41] highlight the multidimensionality and complexity of fibromyalgia. It clearly appears that in addition to the symptomatic picture, the detection of fibromyalgia also requires the assessment of multiple factors including patients’ past history and characteristics, impact of fibromyalgia on patients’ daily life, and situations affecting patients’ condition. Even though the corresponding questions in FibroDetect were not found to be of primary importance in the detection of fibromyalgia patients in the validation study, they most certainly enable patients to express the burden of chronic pain in terms of psychological dimensions, and provide clinicians a more complete picture, and then a better understanding, of their patients’ health status and complaint. They also give meaning to the question included in the FibroDetect score about the patient’s self-recognition. Finally, these questions may be particularly useful for patients with a FibroDetect score of 4 or 5, as they could give clinicians additional information about the patient’s feelings and help them decide whether this patient should be referred to a fibromyalgia specialist or not. Therefore, we suggest keeping these questions in the FibroDetect tool, even though they are not used in the calculation of the FibroDetect score.

FibroDetect is to our knowledge the only fibromyalgia-specific tool that comprehensively covers all domains that are impacted by fibromyalgia as reported directly by patients: physical aspects as well as other aspects that characterize the condition and could be helpful detection factors (pain fatigue, patients’ personal and medical history, patients’ personality and attitude adopted towards their condition, their symptoms, and impact of the condition on their lives). This quality is probably explained by the emphasis on patients that was made during the development phase of the tool. Such emphasis is missing or only partially treated in the recently designed Fibromyalgia Diagnostic Screen and FiRST measures [25,26]. As for the updated fibromyalgia ACR criteria [6], patients’ perspective is not directly considered as the tool is completed by clinicians.. In addition, the performances of these updated criteria to diagnose fibromyalgia at early stage in primary care settings remain to be assessed. Should the FibroDetect be used as a diagnostic tool, a study with a new adapted design should be conducted.

Despite its simple scoring, the length of the FibroDetect tool may limit use in clinical practice because of time constraints faced by PCPs. A short version of the FibroDetect including only the 6 fibromyalgia-physical related burden questions and the self-recognition question used for the scoring could be considered. However, this version would require to be validated prior to being used in clinical practice. The validation study was conducted in France and Germany only. Probably due to the lack of recognition of the disease in the UK on the one hand and recruitment difficulties on the other, validation was not possible in the UK. Therefore, if the tool is to be used in the UK, a preliminary validation in the UK population will be required. In addition, further real life longitudinal studies would be worthwhile to consolidate the validation results of the present study and demonstrate the benefits and usefulness of the tool in primary care settings.

The FibroDetect is available in the form of a four-page leaflet, a user-friendly format particularly well adapted to the context of clinical practice where it can be easily administered to patients during consultation, after or before clinical examination. It is completed in less than 10 minutes. Moreover, this tool can be an effective and standardized method of communication between patients and doctors by facilitating the dialogue and exchange of information. Indeed, the tool can help patients to explain their symptoms and address their needs, and help physicians interpret patients’ complaints and recognise fibromyalgia signs at the same time. The use of such a tool during consultation will not only improve the quality of care in general practice but will also increase patients’ and doctors’ satisfaction and improve patients’ self-esteem and well-being [42].

## Conclusions

The FibroDetect is a robust tool to screen for potential fibromyalgia patients among patients who present to their PCPs with chronic widespread pain and/or fatigue as major complaint. The tool can be used as a surrogate screening classification to ACR criteria for fibromyalgia in primary care settings. It is validated, and is available in French, German, and UK English. By comprehensively assessing the multiple physical and psychological domains and symptoms associated with fibromyalgia, the FibroDetect screening tool will allow a reduction in the delay before diagnosis and appropriate referral to specialists, ensuring access to the best available care. Detection of fibromyalgia is rendered all the more challenging because health professionals are often sceptical regarding the existence of this condition. One advantage of a standardized detection of fibromyalgia, as proposed by the FibroDetect screening tool, is to give legitimacy to the patient’s feedback, which is essential in this controversial pathological condition.

The measure will be accessible to interested researchers at the following website: www.pfizerpatientreportedoutcomes.com.
